# Ankylosing spondylitis with thoracic OPLL and OYL requiring multiple surgeries: A case report

**DOI:** 10.1016/j.ijscr.2025.112127

**Published:** 2025-10-27

**Authors:** Akinori Tani, Kazumasa Nakamura, Katsunori Fukutake, Hiroshi Takahashi, Akihito Wada

**Affiliations:** Department of Orthopedic Surgery, Toho University School of Medicine, Tokyo, Japan

**Keywords:** Ankylosing spondylitis, Ossification of the posterior longitudinal ligament, Ossification of the yellow ligament, Kyphosis, Multiple surgeries, Case report

## Abstract

**Introduction:**

We encountered a rare and complex case of severe kyphosis resulting from ankylosing spondylitis (AS) in conjunction with ossification of the posterior longitudinal ligament (OPLL) and the yellow ligament (OYL), which necessitated multiple surgical interventions.

**Presentation of case:**

The patient was a 45-year-old male with a high body mass index and severe thoracolumbar rigid kyphosis caused by AS. Given the significant degree of the pelvic incidence minus lumbar lordosis (PI-LL) mismatch, a two-stage L2 and L4 pedicle subtraction osteotomy (PSO) was planned, extending from T8 to the pelvis. At that juncture, although concomitant OPLL and OYL at the thoracic spine were observed, these were excluded from the surgical plan, as there were no neurological symptoms before surgery. On the fifth postoperative day following a two-stage surgery, motor weakness and paresthesia in the lower extremities manifested. Based on the neurological findings, a diagnosis of compressive thoracic myelopathy caused by OPLL/OYL at T4–8 level was made, and extensive laminectomy with extended thoracic fusion up to T3 was successfully performed.

**Discussion:**

In retrospect, considering the risks associated with frequent surgery, a single-stage double-level PSO might have been preferable alternative. Furthermore, preparations could have been made for additional thoracic decompression and fusion on a standby basis in case thoracic myelopathy developed due to OPLL/OYL.

**Conclusion:**

The rapid development of thoracic myelopathy following a double-level PSO at the lumbar spine, in the presence of AS and concomitant thoracic OPLL/OYL, despite the presence of ankylosing spine and apparent loss of mobility, was not predicted.

## Highlights

• A complex case of severe kyphosis due to AS with thoracic OPLL and OYL.
• Surgical planning was complicated by obesity, complications, and severity of kyphosis.
• Spinal cord palsy can occur at an ankylosing site above the instrumented fusion.

## 1 Introduction

Ankylosing spondylitis (AS) is a chronic progressive autoimmune disease that often causes a rigid kyphosis deformity. AS is typically diagnosed according to the modified New York criteria and is strongly associated with the HLA-B27 gene. Standard treatment options for AS include nonsteroidal anti-inflammatory drugs, physiotherapy, and tumor necrosis factor -α inhibitors, while surgical correction is considered in severe deformities or advanced neurological involvement [1]. The prevalence of AS shows a different global distribution, strongly associated with HLA-B27 positivity, with higher rates in Northern European and certain Asian populations but relatively lower rates in Japan, with an incidence of 2.6 per 100,000 births [1,2].

In contrast, OPLL and OYL are degenerative spinal ligament ossification (SLO) disorders, usually treated conservatively but often requiring surgical decompression and stabilization when neurological deterioration occurs. The prevalence of ossification of the posterior longitudinal ligament (OPLL) and ossification of the yellow ligament (OYL) in the Japanese population is higher than in Caucasians and South Africans [3].

Although AS and SLO both involve ectopic ossification of the spine, the two diseases are completely different. The prevalence of their coexistence has been documented to range from 2 % to 29 %, contingent on the patient's race and geographical location. Tsuyama et al. reported that the prevalence of OPLL/OYL in conjunction with AS in Japan was only 2 % [4]. On the other hand, Ramos-Remus et al. reported the prevalence in Mexico City was 29 % [5]. To the best of our knowledge, there are only three published case reports of surgical treatment of a patient with thoracic myelopathy due to SLO (one OPLL and two OYL) in combination with AS [[6], [7], [8]]. This report describes a case of severe kyphosis deformity caused by AS with thoracic OPLL and OYL, which required multiple surgical procedures and presented considerable therapeutic challenges. This case report has been reported in line with the SCARE guideline [9].

## 2 Case presentation

A 45-year-old male first noticed kyphosis at age 24, and subsequently experienced gradual progression of low back pain and kyphosis. He was diagnosed with AS at 39, at which time treatment with salazosulfapyridine was initiated and continued; however, kyphosis gradually progressed, and he was experiencing difficulty with ambulation. Maintaining a horizontal gaze during ambulation was only feasible for a few minutes with maximum neck extension. He also had a five-year history of diabetes mellitus.

A physical examination at age 18 indicated height 187 cm, weight 120 kg, and body mass index (BMI) 34.3 kg/m^2^. He had a significant kyphosis deformity, but no abnormal neurological findings.

**Fig. 1:**
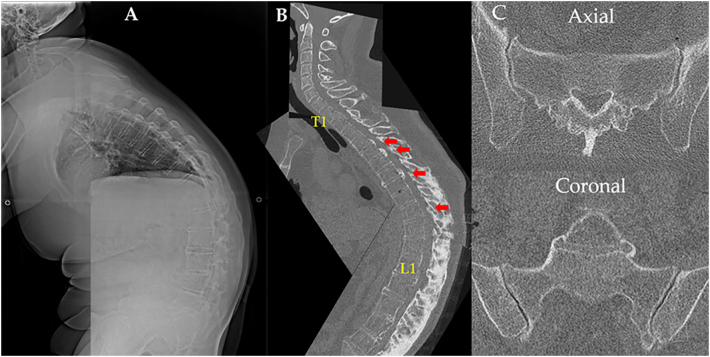
A) Standing sagittal radiograph at the initial examination showing significant kyphosis of the entire thoracolumbar spine and so-called “bamboo spine”. B) Sagittal reconstructed computed tomography at the initial examination showing abnormal hyperostosis in the anterior longitudinal ligaments and massive ossification of the supraspinous ligament. Red arrows indicate segmental type OPLL at T4–8/L1–2 and OYL at T10–11. C) CT scan of the sacroiliac joints showing bilateral narrowing of the joint gap and osteosclerosis. (For interpretation of the references to colour in this figure legend, the reader is referred to the web version of this article.)

**Fig. 2:**
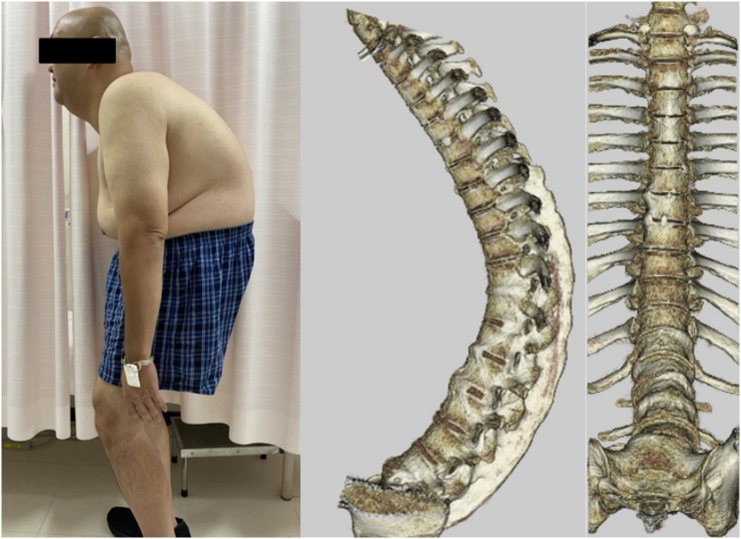
Lateral view just before surgery and 3DCT showing ossification of the anterior longitudinal ligament and supraspinous ligament, resulting in rigid severe kyphosis.

A standing lateral radiograph revealed a notable degree of kyphosis throughout the thoracolumbar spine. The sagittal spinopelvic parameters were: LL = −32°, PI = 61°, PI–LL = 93°, pelvic tilt (PT) = 84°, thoracic kyphosis (TK) = 54°, and sagittal vertical axis (SVA) = 340 mm. Lateral sagittal reconstructed computed tomography (CT) of the thoracolumbar spine revealed abnormal vertebral hyperostosis (“bamboo spine”) and segmental type OPLL at T4–8/L1–2 in conjunction with OYL at T10–11 (Fig. 1A, B). CT of the sacroiliac joints showed bilateral narrowing of the joint spaces and osteosclerosis (Fig. 1C). The patient was unable to undergo magnetic resonance imaging (MRI) due to his large size and rigid kyphotic posture, which prevented assumption of the required position. Myelography was conducted, but the spinal tap failed due to AS and OYL. 3D-CT showed ossification of the supraspinous ligament, resulting in severe kyphosis (Fig. 2).

**Fig. 3:**
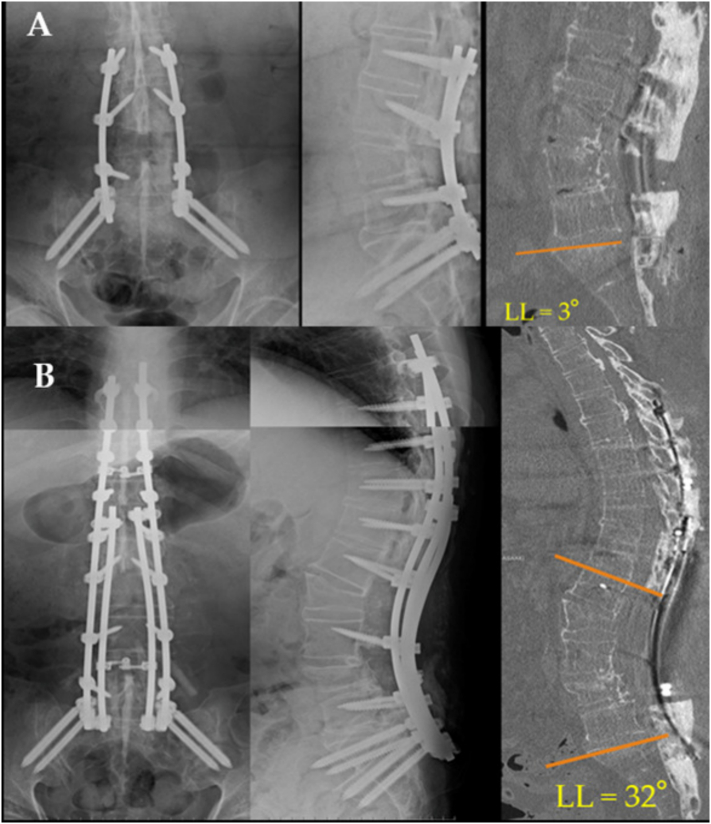
A) Frontal and lateral radiographs after the 1st stage operation showing increased lumbar lordosis to 3° by L4 PSO and posterior instrumentation from L2 to the pelvis. B) Frontal and lateral radiographs after the 2nd stage operation showing increased lumbar lordosis to 32° by L2 PSO and posterior instrumentation from Th8 to the pelvis.

The HLA-B27 gene test was negative, but the patient was diagnosed with AS using the modified New York criteria (Table 1), consistent with both clinical symptoms and radiological findings. After careful consideration, we determined that the primary pathology was severe kyphosis of the rigid lumbar spine due to AS. Given the PI–LL mismatch of 93°, a single vertebral osteotomy was deemed insufficient; instead, two-stage double-level pedicle subtraction osteotomy (PSO) was planned. While concomitant thoracic OPLL and OYL were identified, they were excluded from the plan because there were no neurological symptoms preoperatively. In the initial phase, L4 PSO with posterior L2–iliac fusion was conducted, resulting in LL improvement to 3° (Fig. 3A). After a one-week interval, the second stage, L2 PSO with posterior T8–iliac fusion was performed, resulting in improvement of LL to 32° (Fig. 3B).

**Table 1:** Modified New York Criteria for ankylosing spondylitis (AS) (1984).

Criteria	Description
Clinical criteria	(a) Low back pain and stiffness for more than 3 months, which improves with exercise, but is not relieved by rest (b) Limitation of motion of the lumbar spine in both the sagittal and frontal planes (c) Limitation of chest expansion relative to normal values adjusted for age and sex
Radiological criterion	Sacroiliitis grade ≥ 2 bilaterally or grade 3–4 unilaterally Grade 0 = normal Grade 1 = suspicious Grade 2 = sclerosis, some erosions Grade 3 = severe erosion, widening of the joint space, some ankylosis Grade 4 = complete ankylosis

Definite AS is present if the radiological criterion is associated with at least 1 clinical criterion.

**Fig. 4:**
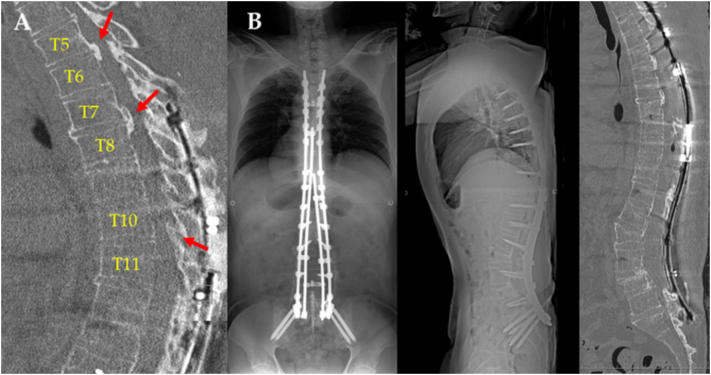
A) CT scan on postoperative day 5, just after onset of neurological symptoms, showing OPLL and OYL at T5/6 and T7/8, and OYL at T10/11 (arrows). B) Extensive laminectomy and extended fixation up to T3 were finally performed.

On postoperative day (POD) 5, the patient exhibited motor weakness of the lower extremities (Frankel grade C), accompanied by numbness and paresthesia below the navel, upon standing and initiating ambulation. Despite the potential involvement of the conus medullaris involvement related to the L2 PSO was considered, the profound sensory loss and severe numbness extending from the periumbilical trunk, suggesting T10 dermatome involvement, downward could not be fully explained by conus impairment alone. Intraoperative spinal cord monitoring during the second-stage surgery showed no significant decrease in both sensory and motor evoked potentials, and during the postoperative period up to POD 5, while the patient remained on strict bed rest, there was no motor weakness, truncal sensory loss, or numbness. These findings suggested that the paraplegia was caused by thoracic OPLL and OYL. Due to his size, MRI could not be performed; thus, only plain CT of the thoracic spine was available (Fig. 4A). An urgent, extensive laminectomy was therefore performed from T5 to T9, with de-kyphosis stabilization extending to T3 (Fig. 4B).

After the third procedure, there was gradual improvement in symptoms associated with thoracic myelopathy and paralysis. However, on POD 10, fever developed with elevated CRP, causing surgical site infection (SSI). *Candida albicans* was identified as the causative organism and wound debridement was performed under general anesthesia.

Three years after the most recent procedure, the patient still experiences spasticity in his left lower extremity but can walk with a cane.

## 3 Discussion

This case report highlights several challenging clinical issues. First, there is the potential for thoracic cord palsy to occur between the segment of long caudal instrumented correction and fusion and the non-surgical site when there is intraspinal ossification at the junction, even though the spinal column apparently ankylosed. In this case, the patient was a large man with a severe kyphotic deformity due to the combination of AS, OPLL, and OYL. Although conus medullaris impairment related to PSO was considered, the severe sensory disturbance extending from the periumbilical trunk downward could not be explained by conus involvement alone. Instead, the presentation was more consistent with thoracic myelopathy caused by ossified lesions at cranial non-fused thoracic levels. Second, considering the invasive nature of corrective surgery in adult patients with severe thoracolumbar kyphosis using the PSO technique, it was inappropriate including neurologically asymptomatic thoracic OPLL/OYL in the surgical plan. As a result, the patient developed acute thoracic myelopathy immediately following ambulation, necessitating further surgical intervention for the upper thoracic spine. Thoracic cord compression could not be confirmed preoperatively, making it difficult to decide whether decompression and fusion to the T3 level should be included in the second surgery.

Previous studies have suggested that longer fusion in AS cases is associated with an increased risk of proximal junctional kyphosis and fracture (PJK/PJF). In posterior osteotomy in patients with AS, Wang et al. [10] found that the incidence of PJK was significantly higher in patients with fusion above the T9 vertebral level (36 %, 9/25) than in those with fusion below T9. Ikegami et al. [11] reported paralysis resulting from PJF in the case of an elderly female with diffuse idiopathic skeletal hyperostosis (DISH), with PJF occurring at the ankylosing site above the uppermost instrumented vertebrae after long-segment instrumentation fusion. Current case is the first to demonstrate that postoperative thoracic spinal cord paralysis can occur due to SLO within the spinal canal, rather than as a result of postoperative PJF.

Finally, there was a risk of surgical invasiveness. We planned a two-stage double-level PSO, given the difficulty of correcting a single vertebral osteotomy with a large PI–LL mismatch and our concerns regarding skin problems and the risk of SSI due to diabetes mellitus, which could result from prolonged surgery. Woquan et al. reported that single-stage double-level PSO could be performed relatively safely in AS cases with SVA >15 cm or kyphosis angle (T1–S1) >90° [12]. In contrast, Han et al. found that prolonged prone surgery in patients with severe obesity results in increased blood loss and an elevated risk of perioperative complications, including postoperative visual disturbance, deep vein thrombosis, pulmonary embolism, lateral femoral cutaneous neuropathy, and pressure sores [13]. Thus, addition of extensive decompression and fixation to the upper thoracic spine in the second-stage surgery was expected to pose a considerable risk. In retrospect, a single-stage double-level PSO might have been preferable to reduce cumulative invasiveness, with preparations for additional thoracic decompression and fusion if neurological deterioration occurred.

Although rare, the coexistence of ankylosing spondylitis with OPLL and OYL represents a clinically significant condition. Epidemiologically, the overlap of two geographically distinct diseases may be underestimated, particularly in East Asian populations where OPLL and OYL are prevalent. Pathogenetically, shared mechanisms of abnormal ossification are suspected but remain to be elucidated. Clinically, these patients are at particularly high risk for spinal cord compression and neurological decline, and surgical management is often complex. Reporting additional cases and analyzing their clinical, radiological, and genetic characteristics may help clarify the underlying associations and improve management strategies for this challenging condition.

## 4 Conclusion

We experienced a rare case of AS concomitant with thoracic OPLL and OYL. This case presented a significant challenge in treatment planning due to unexpected myelopathy at the junction of a long-fused segment, despite spinal ankylosis and apparent loss of mobility. This case shows that paraplegia can occur at an ankylosing site above the uppermost instrumented vertebra after long-segment spinopelvic fusion for AS with thoracic OPLL and OYL.

## Ethical approval

Institutional Review Board approval was not required because the study is a descriptive analysis of an individual case. The patient was informed that data from this case would be submitted for publication and gave his written consent.

## Funding

Institutional sources only.

## Author contribution

Akinori Tani: Principal investigator and primary author: Primary drafting and revision of the manuscript.

Kazumasa Nakamura: Senior author and clinical advisor: performed PSO surgery.

Katsunori Fukutake: Co-author: Data analysis and discussion.

Hiroshi Takahashi: Co-author: Literature review, editing the manuscript.

Akihito Wada: Senior author and clinical advisor: Performed all surgeries and diagnosed the patient, contributing expert insight into the clinical aspects of the case. Provided detailed descriptions of the diagnostic process, surgical procedure, and overall clinical management and postoperative follow-up. Supervised and edited the manuscript.

## Conflict of interest statement

The authors have nothing to disclose with regard to publication of this case report.

## Guarantor

Akihito Wada

## Research registration number

Not applicable

## Declaration of competing interest

The authors declare that they have no known competing financial interests or personal relationships that could have appeared to influence the work reported in this paper.
